# Multimodal PPG-Based Arrhythmia Detection Using a CLIP-Initialized Multi-Task U-Net and LLM-Assisted Reporting

**DOI:** 10.3390/s26082316

**Published:** 2026-04-09

**Authors:** Youngho Huh, Minhwan Noh, Dongwoo Ji, Yuna Oh, Sukkyu Sun

**Affiliations:** 1 Department of Computer Science and Artificial Intelligence, Dongguk University, Seoul 04620, Republic of Korea; yangkos17@dgu.ac.kr (Y.H.); will1125@dgu.ac.kr (D.J.); 2 Division of AI Convergence, Department of Data Science, Dongguk University, Seoul 04620, Republic of Korea; min1026hwan@dgu.ac.kr (M.N.); yuno00@dgu.ac.kr (Y.O.)

**Keywords:** photoplethysmography (PPG), heart rate variability (HRV), clinical information, arrhythmia detection, contrastive learning, large language models (LLMs), retrieval-augmented generation (RAG), explainable AI (XAI)

## Abstract

Photoplethysmography (PPG) has emerged as an attractive modality for non-invasive cardiovascular monitoring due to its low cost, unobtrusive nature, and ubiquity in consumer wearable devices. Despite its potential, existing PPG-based arrhythmia detection systems remain limited in scope: (i) most target only atrial fibrillation, (ii) temporal localization of abnormal segments is rarely provided, and (iii) deep learning models lack explainability, hindering adoption in clinical workflows. We present a comprehensive and fully integrated framework for multi-class arrhythmia detection, segmentation, and explainability based on PPG waveforms, Heart Rate Variability (HRV), and structured clinical metadata. The proposed system introduces a CLIP-style contrastive learning module aligning PPG waveforms with clinical variables and rhythm-state textual descriptions using BioBERT; a multitask U-Net architecture performing 4-class classification and 1D segmentation; a Retrieval-Augmented Generation (RAG) pipeline leveraging Gemini Flash large language models to produce guideline-grounded diagnostic reports; and a real-time Streamlit-based web platform supporting inference, visualization, and database storage. The system significantly improves classification accuracy (from 86.27% to 91.19%) and segmentation Dice (from 0.5815 to 0.7167). These results demonstrate the feasibility of a robust, multimodal, and explainable PPG-based arrhythmia monitoring system for real-world applications.

## 1. Introduction

Photoplethysmography (PPG) is an optical sensing modality that measures peripheral blood volume changes using a light-emitting diode (LED) and photodetector [1]. PPG signals are widely used to monitor oxygen saturation, heart rate, and vascular properties and they are foundational to modern wearable devices such as smartwatches and fitness trackers [2,3]. Due to its non-invasive nature, ease of acquisition, and low hardware complexity, PPG has gained significant attention as a potential alternative or complement to electrocardiography (ECG) for cardiovascular monitoring, particularly in ambulatory and remote healthcare settings [4].

### 1.1. Limitations of Current PPG-Based Arrhythmia Detection

Despite its widespread availability, PPG-based arrhythmia detection faces several challenges [5]:

- Narrow detection scope: Contemporary consumer-grade wearable devices are predominantly optimized for Atrial Fibrillation (AF) screening [6], while offering limited capabilities for detecting Bradycardia or Tachycardia.
- Lack of temporal localization: Segment-level classification without timestamp localization limits clinical usefulness [5,6].
- Class-within variability: Even within the same arrhythmia class (AF, B, and T), PPG waveforms present highly diverse temporal patterns [5].
- Noise susceptibility: Motion artifacts, variable contact pressure, and perfusion variability degrade reliability [7].
- Limited generalization of supervised models: Conventional deep learning approaches struggle to learn stable, patient-invariant representations of PPG rhythm dynamics.
- Black-box deep learning models: Clinicians require transparent, guideline-aligned reasoning, rather than just predictions [8].

Thus, improving robustness, interpretability, and multi-class capability remains essential to facilitate clinical adoption.

### 1.2. Related Work and Comparison Motivation

Prior PPG-based arrhythmia studies primarily focus on binary AF screening [6] or limited multi-class settings [5], often lacking precise temporal localization and granular performance characterization under significant class imbalance. Moreover, many works rely on private datasets, which limits reproducibility and direct benchmarking. Motivated by these gaps, we provide (i) multi-class detection (Normal/AF/B/T), (ii) per-sample segmentation for temporal localization, and (iii) a comprehensive evaluation of classification performance using class-specific metrics—including precision, recall, and F1-score—to rigorously assess model reliability across both prevalent and rare arrhythmia subtypes.

### 1.3. Contributions

**Figure 1:**
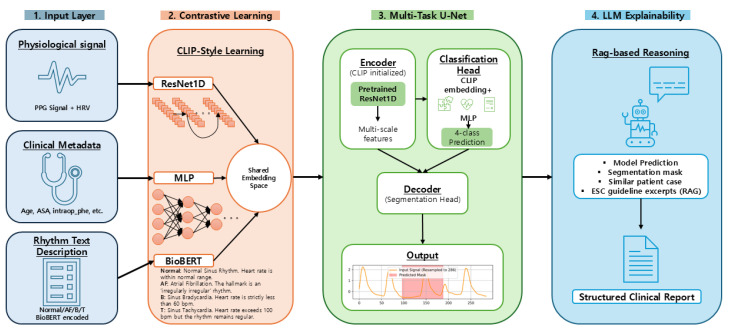
Overview of the proposed multimodal PPG-based arrhythmia detection framework integrating contrastive pretraining, multi-task learning, and LLM-based explainability. Physiological inputs consisting of PPG segments and HRV features, structured clinical metadata, and rhythm-specific textual descriptions are first aligned through CLIP-style contrastive learning, where waveform, clinical, and text representations are projected into a shared embedding space. The pretrained encoder is then reused to initialize a multi-task 1D U-Net that simultaneously performs four-class rhythm classification (Normal, Atrial Fibrillation, Bradycardia, and Tachycardia) and temporal segmentation of abnormal intervals. The model outputs, together with similar-patient retrieval results and guideline excerpts, are provided to a Retrieval-Augmented large language model (LLM), which generates structured and clinically grounded diagnostic reports. This architecture enables unified representation learning, temporal localization, and interpretable clinical reasoning within a single end-to-end system.

To address these challenges, we propose an end-to-end framework integrating multimodal contrastive pretraining, segmentation-assisted classification, and guideline-grounded interpretability (Figure 1).

- Multimodal Contrastive Alignment for Arrhythmia Representation: We propose a CLIP-style pretraining strategy that synergizes 1D PPG waveforms with structured clinical variables—including key HRV features such as HR, SDNN, RMSSD, and pNN50—and BioBERT-based textual descriptions [9,10]. By aligning these disparate modalities in a shared embedding space, the model learns robust physiological representations that bridge the gap between raw signal patterns and clinical diagnostic language.
- Multitask U-Net Architecture for Granular Diagnostics: We implement a multitask U-Net framework designed for simultaneous 4-class arrhythmia classification (Normal, AF, Bradycardia, Tachycardia) and per-sample temporal segmentation. This architecture facilitates precise temporal localization of arrhythmic events, achieving an accuracy of 91.2%, representing an improvement over standalone signal processing models by leveraging the learned multimodal embeddings [11].
- Guideline-Grounded Explainable AI (XAI) via RAG: To enhance clinical trustworthiness, we introduce a Retrieval-Augmented Generation (RAG) pipeline utilizing the Gemini LLM [12]. This system generates assistive, guideline-grounded interpretative summaries by grounding model predictions in retrieved evidence from the European Society of Cardiology (ESC) guidelines and similar historical patient cases, providing interpretable justifications for each diagnosis [13].
- Real-Time Clinical Decision Support System: We develop and deploy a comprehensive Streamlit-based interface that enables real-time PPG inference, waveform visualization, and automated report generation. The system is integrated with a database for longitudinal patient tracking, demonstrating the practical feasibility of deploying complex multimodal AI in ambulatory and perioperative settings.

To our knowledge, this is the first fully integrated framework combining contrastive learning, multitask modeling, similar-patient retrieval, and large language model reasoning for PPG arrhythmia analysis.

## 2. Materials and Methods

### 2.1. Dataset Description from VitalDB

The dataset used in this study was derived from VitalDB [14], a publicly available high-resolution perioperative biosignal database collected from 6388 surgical patients at Seoul National University Hospital. VitalDB provides synchronized waveform recordings, including electrocardiography (ECG), photoplethysmography (PPG), arterial blood pressure (ABP), capnography, and ventilator parameters, along with more than 60 structured clinical variables. All data are fully de-identified and were collected under prior institutional review board (IRB) approval.

In this study, we utilized the Lead II ECG waveform and the PPG waveform (PLETH channel) for arrhythmia analysis. To ensure clinical homogeneity and reliable physiological signal interpretation, explicit inclusion and exclusion criteria were applied as follows:

- Inclusion criteria:
  – Availability of both Lead II ECG and PPG waveforms: Required to enable ECG-based rhythm verification and PPG-based arrhythmia modeling.
  – Adult patients aged ≥ 18 years: Pediatric patients exhibit distinct cardiovascular dynamics and PPG morphology, which may confound model generalization.
  – Surgical duration ≥ 2 h: Ensures sufficient continuous waveform data for stable rhythm analysis and segmentation.
  – Cases performed under general anesthesia: General anesthesia minimizes motion artifacts and autonomic fluctuations, improving signal stability.
- Exclusion criteria:
  – Cardiovascular or major vascular surgery: These procedures often involve direct cardiac manipulation or extracorporeal circulation, which may induce non-physiological rhythm patterns unrelated to intrinsic arrhythmia.
  – Organ transplantation surgery: Associated with severe hemodynamic instability and pharmacologic interventions that can distort PPG morphology.
  – Body weight ≤ 35 kg or ≥120 kg: Extreme body mass may significantly affect peripheral perfusion and pulse wave propagation characteristics.
  – Device malfunction, signal dropouts, or flatline segments: Excluded to prevent corrupted waveform segments from biasing peak detection and segmentation training.

After applying the above criteria, 2379 patients remained for further analysis. Subsequently, signal-quality screening was conducted based on waveform continuity and peak detectability. For each patient, the distribution of R–R intervals derived from the Lead II ECG was analyzed. Patients exhibiting a high proportion of excessively long or short R–R intervals relative to their individual mean were categorized as suspected arrhythmia candidates.

Among these, 25 patients were confirmed by a board-certified cardiologist to have clinically meaningful arrhythmias, including Atrial Fibrillation, Bradycardia, and Tachycardia. These patients formed the arrhythmia cohort. Additionally, 10 patients with exclusively normal sinus rhythms throughout the recording period were selected to form the normal control cohort in order to partially mitigate class imbalance.

The final dataset was then segmented and processed as described in the subsequent sections.

**Figure 2:**
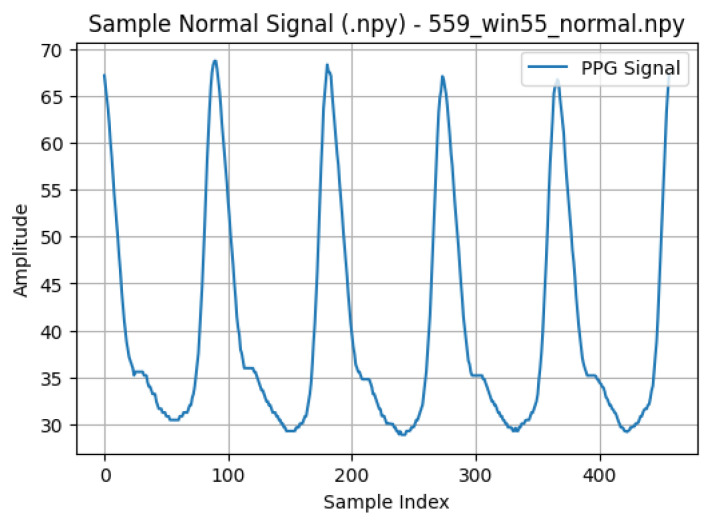
Example of a normal PPG segment used in this study. The waveform shows stable amplitude and nearly periodic beat-to-beat intervals.

**Figure 3:**
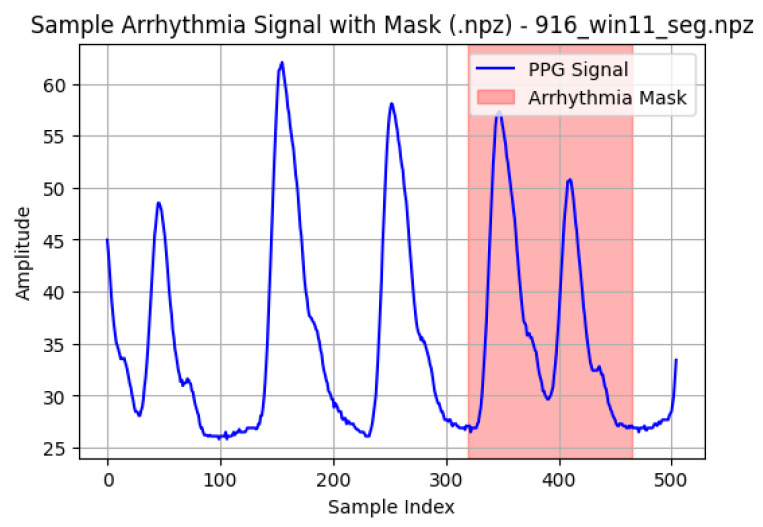
Example of an arrhythmia PPG segment with the corresponding abnormal mask (shaded region). The highlighted interval indicates the time span labeled as arrhythmic.

**Figure 4:**
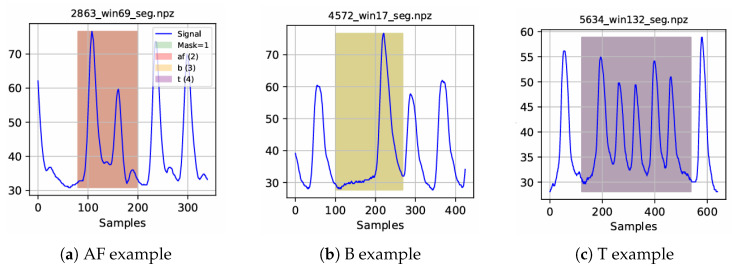
Representative arrhythmia PPG segments for (**a**) Atrial Fibrillation (AF), (**b**) Bradycardia, and (**c**) Tachycardia. Each shaded region corresponds to the abnormal interval used to generate the segmentation mask.

A representative normal PPG segment is shown in Figure 2, demonstrating stable pulse morphology and periodicity. Figure 3 illustrates a sample arrhythmia segment with its corresponding abnormal mask generated from ECG-derived annotations. To highlight rhythm-specific waveform characteristics, examples of Atrial Fibrillation, Bradycardia, and Tachycardia segments are provided in Figure 4.

### 2.2. PPG Sensor and Acquisition Details

The PPG waveforms analyzed in this study were obtained from the publicly available VitalDB database [14], which provides high-resolution perioperative biosignals recorded in routine operating room monitoring at Seoul National University Hospital. In VitalDB, PPG is stored as a plethysmography (PLETH) waveform acquired from standard bedside patient monitors (Philips IntelliVue series), under general anesthesia where patient motion is minimized and probe contact is relatively stable (Table 1).

- Acquisition setting and measurement mode: VitalDB does not explicitly provide probe placement and optical configuration in its public metadata. However, intraoperative pulse oximetry in tertiary hospitals is typically measured using a transmission-mode finger probe connected to bedside monitors [3]. Therefore, when a parameter is not explicitly specified by VitalDB, we report acquisition characteristics consistent with standard intraoperative pulse oximetry practice and clearly distinguish them from study-specific preprocessing (Section 2.6).
- Sampling frequency and recording duration: The original VitalDB waveforms are recorded at high sampling rates in the perioperative setting (commonly 500 Hz across waveform tracks in the VitalDB ecosystem), and downsampling is frequently applied for downstream analysis in public benchmark pipelines. In this study, we downsampled the PPG waveform to 100 Hz to reduce computational burden while preserving rhythm-related temporal patterns. Each patient record in our screened cohort contained approximately 9–10 min of continuous PPG, although the exact duration varies by surgical case and recording availability.
- Signal units and device-dependent scaling: The pleth waveform amplitude is provided in device-dependent arbitrary units (a.u.) without absolute physiological calibration. Thus, absolute amplitude values should be interpreted as relative changes rather than calibrated volumetric measures.
- Unavailable sensor specifications in public metadata: Hardware specifications such as optical wavelength, photodiode sensitivity, analog front-end gain, and manufacturer-calibrated sensitivity are not provided in the public VitalDB metadata. We therefore report the clinically relevant acquisition context (monitor type, intraoperative setting, typical transmission-mode finger probe usage, sampling frequency, and recording duration) and explicitly document all preprocessing steps applied prior to model input (Section 2.6) to ensure reproducibility and transparency.

### 2.3. Patient-Level Split and Data Ratio

To avoid information leakage, all splits were performed at the patient level [15]. The final splits were as follows:

- Training Data
  22 patients (14 arrhythmia + 8 normal), 948 segments (445 arrhythmia + 503 normal).
- Validation Data
  8 patients (6 arrhythmia + 2 normal), 158 segments (70 arrhythmia + 88 normal).
- Test Data
  5 arrhythmia patients with mixed rhythms, 386 segments.

Overall, the patient-level ratio is approximately 22:8:5 for train/validation/test. The segment counts are reported above because arrhythmia prevalence and peak-based subdivision create unequal numbers of segments per patient.

### 2.4. Clinical Variables and Rationale

Thirteen structured clinical variables were used, including the following:

- Age, sex, body mass index (BMI), and operation duration.
- Sodium, potassium, and chloride.
- Blood urea nitrogen and creatinine.
- Diabetes mellitus and hypertension status.
- Emergency operation flag.
- Intraoperative vasopressor counts (ephedrine and phenylephrine).

The 13 clinical variables incorporated into our model were selected based on their established physiological relevance to PPG morphology and arrhythmia risk. Demographic factors such as age, sex, and body mass index (BMI) influence arterial stiffness, autonomic regulation, and peripheral perfusion, all of which can alter waveform characteristics. Electrolyte markers, including preoperative sodium and potassium, were included due to their direct effects on cardiac electrophysiology and their well-known association with arrhythmogenesis [16].

Renal function indicators (preoperative BUN and creatinine) capture volume status and metabolic imbalance, both of which can modulate PPG amplitude and variability. Chronic comorbidities such as diabetes mellitus and hypertension were selected for their impact on autonomic tone, microvascular perfusion, and arterial compliance, each contributing to waveform variability relevant to arrhythmia detection.

To account for perioperative physiological stress, we included operation duration and emergency operation status, which reflect intraoperative instability that often coincides with arrhythmic events. Finally, intraoperative vasopressor administration (ephedrine and phenylephrine) was incorporated as a proxy for acute hemodynamic perturbations, since vasoactive drugs significantly alter peripheral vascular tone and thus influence PPG morphology [3].

Together, these variables provide a multidimensional representation of patient physiology, complementing raw waveform features and enabling the model to learn clinically meaningful relationships between PPG signals and arrhythmic states.

### 2.5. PPG Segmentation and Labeling

#### 2.5.1. Primary Segmentation: 60 s Windows

PPG signals were sampled at 100 Hz and initially segmented into non-overlapping 60 s windows (6000 samples), providing clinically meaningful context and sufficient rhythm variability.

#### 2.5.2. Secondary Segmentation: Peak-Based Subdivision

Heart rate varies substantially across patients (40–120 peaks/min). To normalize rhythm granularity, each 60 s window underwent

- Adaptive systolic peak detection [2].
- Peak count estimation.
- Subdivision into shorter segments containing approximately five peaks each on average.

The resulting segments ranged from 286 to 785 samples in length.

#### 2.5.3. Label Assignment and Segmentation Masks

Rhythm labels (Normal, AF, Bradycardia, Tachycardia) were assigned using ECG-based annotations from VitalDB. Binary segmentation masks were generated as

(1) $$m\left(i\right)=\left\{\begin{array}{cc} 1 & (\mathrm{abnormal}\;\mathrm{rhythm}\;\mathrm{at}\;\mathrm{index}\;i)\\ 0 & \left(\mathrm{otherwise}\right) \end{array}\right.\;$$

Segments containing mixed rhythms were excluded to avoid ambiguous supervision.

### 2.6. Signal Preprocessing Pipeline

Because preprocessing can significantly alter PPG morphology and fiducial points, we explicitly describe the pipeline used prior to model input. The pipeline is designed to preserve rhythm-related morphology while standardizing amplitude and length.

#### 2.6.1. Quality Control

We excluded segments with (i) near-flatline behavior, (ii) extremely low variability, or (iii) insufficient peak detectability. Concretely, segments were rejected if the standard deviation fell below a small threshold or if fewer than three valid systolic peaks were detected in the parent 60 s window.

#### 2.6.2. Length Standardization via Interpolation

All segments were resampled to a fixed length of 286 (minimum observed length) using linear interpolation for waveform signals:

(2) $$\tilde{x}\left(u\right)=x\left(t_{0}\right)+\left(x\left(t_{1}\right)-x\left(t_{0}\right)\right)\cdot \frac{u-t_{0}}{t_{1}-t_{0}}\;$$

Segmentation masks were resampled using nearest-neighbor interpolation to preserve boundary sharpness:

(3) $$\tilde{m}\left(u\right)=m\left(\mathrm{round}\left(t\left(u\right)\right)\right)$$

#### 2.6.3. Z-Score Normalization

Each segment was normalized using

(4) $$x_{\mathrm{norm}}\left(i\right)=\frac{x(i)-\mu }{\sigma }\;$$

where μ and σ are the segment-wise mean and standard deviation. This mitigates amplitude variations caused by perfusion changes or sensor contact pressure.

### 2.7. Heart Rate Variability Features

To effectively characterize the physiological differences between the four arrhythmia classes (Normal, AF, Bradycardia, and Tachycardia), we extracted four key time-domain HRV features from the PPG-derived P-P intervals. These features were selected to provide a balanced representation of both absolute heart rate levels and beat-to-beat irregularity [16].

1. Heart Rate (HR): Calculated as the number of beats per minute (BPM). This is the definitive feature for identifying heart rate-based abnormalities; specifically, HR<60 bpm is indicative of Bradycardia (B), while HR>100 bpm suggests Tachycardia (T).
  (5) $$\mathrm{HR}=\frac{60}{\bar{RR}}=\frac{60\times N}{\sum_{i=1}^{N}RR_{i}}$$
2. SDNN (Standard Deviation of NN intervals): This metric represents the overall variability of the heart rate during the recording period. It reflects the total autonomic influence on the cardiac cycle.
  (6) $$\mathrm{SDNN}=\sqrt{\frac{1}{N-1}\sum_{i=1}^{N}{\left(RR_{i}-\bar{RR}\right)}^{2}}$$
3. RMSSD (Root Mean Square of Successive Differences): This feature quantifies short-term beat-to-beat variations. High RMSSD values are a hallmark of the ‘irregularly irregular’ rhythm seen in Atrial Fibrillation (AF).
  (7) $$\mathrm{RMSSD}=\sqrt{\frac{1}{N-1}\sum_{i=1}^{N-1}{\left(RR_{i+1}-RR_{i}\right)}^{2}}$$
4. pNN50: The percentage of successive P-P intervals that differ by more than 50 ms. Similar to RMSSD, pNN50 is a robust indicator of high-frequency heart rate fluctuations, providing additional evidence for detecting AF.
  (8) $$\mathrm{pNN}50=\frac{\mathrm{count}(|RR_{i+1}-RR_{i}|>50\;\mathrm{ms})}{N-1}\times 100\%$$

### 2.8. Baseline Multi-Task U-Net

**Figure 5:**
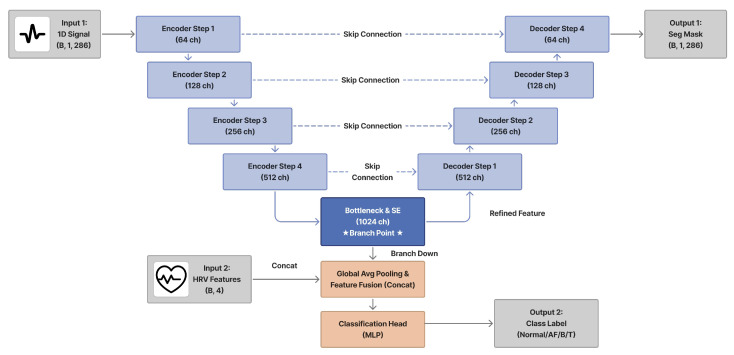
Baseline multi-task 1D U-Net. The encoder–decoder path predicts a segmentation mask for abnormal intervals, while a squeeze-and-excitation (SE) block refines the bottleneck feature, which is then fused with HRV features and passed to a classification head for four-class rhythm prediction.

As a baseline, we implemented a 1D multi-task U-Net that jointly performs arrhythmia segmentation and rhythm classification using only the PPG waveform and HRV features (Figure 5). All input segments are resampled to a fixed length of 286 samples so that both tasks operate on temporally aligned sequences.

#### 2.8.1. Encoder–Decoder Path

The encoder follows a typical U-Net-style hierarchy. An initial DoubleConv block maps the input PPG signal from 1 to 64 channels, followed by four downsampling blocks with channel widths of 128, 256, 512, and 1024. Each down block applies max pooling with stride 2 and a DoubleConv module (two 3×1 convolutions with Group Normalization and ReLU), progressively increasing the receptive field while reducing temporal resolution.

The decoder mirrors this structure with four upsampling blocks. Each block upsamples the feature map by a factor of 2 (linear interpolation), concatenates it with the corresponding encoder feature map via a skip connection, and applies another DoubleConv. A dedicated segmentation head (three 1D convolutional layers with Group Normalization and ReLU) finally maps the 64-channel decoder output to a single-channel abnormal-interval mask $\hat{m}\in R^{286}$.

#### 2.8.2. Bottleneck and SE Block

At the deepest level (1024 channels), we insert a 1D squeeze-and-excitation (SE) block. The SE module performs global average pooling along the temporal dimension, passes the resulting channel descriptor through a two-layer MLP, and generates channel-wise weights in [0,1]. These weights re-scale the bottleneck feature map, emphasizing informative channels before it is fed to both the decoder and the classification branch.

#### 2.8.3. Classification Branch with HRV Fusion

For rhythm classification, the SE-refined bottleneck feature is globally average pooled to obtain a 1024-dimensional waveform embedding. This embedding is concatenated with a 4-dimensional HRV feature vector (heart rate, SDNN, RMSSD, and pNN50), producing a joint representation of waveform morphology and beat-to-beat variability. The concatenated feature is passed through a multilayer perceptron (MLP) with Layer Normalization, ReLU activations, and dropout, yielding logits for the four rhythm classes (Normal/AF/B/T).

#### 2.8.4. Multi-Task Loss

The baseline model is trained end-to-end with a weighted sum:

(9) $$L_{{\mathrm{total}}_{1}}=\alpha \;L_{\mathrm{clf}}+\beta \;L_{\mathrm{seg}},\;$$

where $L_{\mathrm{clf}}$ is a class-weighted focal loss [17] (with an asymmetric penalty to better handle clinically important misclassifications) and $L_{\mathrm{seg}}$ combines BCE and Dice loss [18]. In our experiments, we set α=1.8 and β=0.9 to balance rhythm classification and segmentation performance. This baseline architecture (Figure 5) serves as the reference model when evaluating the effect of contrastive pretraining and multimodal fusion in the proposed CLIP-initialized model.

### 2.9. Contrastive Pretraining: CLIP-BioBERT Framework

**Figure 6:**
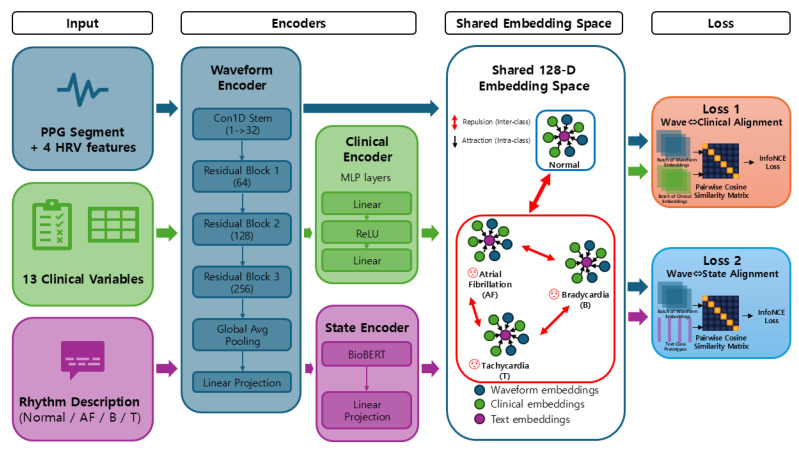
Overview of the CLIP-style contrastive pretraining framework. PPG waveforms are encoded via ResNet1D, clinical variables via an MLP, and rhythm descriptions via BioBERT. All are projected into a shared 128-dimensional space and optimized with InfoNCE losses for wave–clinical and wave–state alignment.

To enrich the representation of PPG morphology and its relationship with patient context, we employed a CLIP-style contrastive pretraining framework [9] (Figure 6).

#### 2.9.1. Waveform Encoder

The waveform encoder consists of a 1D ResNet architecture [19] with four residual blocks. Each block contains two convolutional layers (kernel size = 3, stride = 1), followed by Batch Normalization and ReLU activation, with identity skip connections for residual learning. The architecture progressively increases channel dimensions: 64 → 128 → 256 → 512 channels across the four blocks. After the residual blocks, global average pooling is applied, followed by fully connected layers that project the features to a 128-dimensional latent embedding:

(10) $$z_{w}\in R^{128}$$

#### 2.9.2. Clinical Metadata Encoder

The clinical metadata were encoded using a two-layer multilayer perceptron (MLP). The input consisted of 13 clinical features, comprising nine continuous variables (age, BMI, operation duration, preoperative sodium, potassium, BUN, creatinine, intraoperative ephedrine, and intraoperative phenylephrine) and four binary variables (sex, emergency operation status, diabetes mellitus, and hypertension). Prior to encoding, continuous variables were standardized using z-score Normalization based on the statistics computed from the training set, whereas binary features were encoded as {0, 1}. Missing values were imputed using the median (continuous) or the mode (binary) of the training distribution.

The MLP consisted of two fully connected layers:

- Layer 1: Linea r ( 13 → 64) followed by Batch Normalization and ReLU activation
- Layer 2: Linea r ( 64 → 128)

The output of this encoder, denoted

(11) $$z_{c}\in R^{128}\;$$

provides a compact representation of patient-specific physiological context that complements the PPG waveform features in the contrastive learning framework.

#### 2.9.3. State Encoder (BioBERT)

Textual rhythm descriptions were encoded using BioBERT (biobert-base-cased-v1.1) [10], a transformer model pretrained on large-scale biomedical corpora. For each of the four rhythm classes (Normal, Atrial Fibrillation, Bradycardia and Tachycardia), clinically consistent textual definitions were constructed:

- Normal
  “Normal Sinus Rhythm (NSR). Heart rate is within normal range (60–100 bpm).”
  “Regular and consistent beats with stable P-P intervals.”
- Atrial Fibrillation
  “Atrial Fibrillation. The hallmark is an ‘irregularly irregular’ rhythm.”
  “P waves are completely absent and replaced by chaotic fibrillatory waves.”
  “P-P intervals vary unpredictably.”
- Bradycardia
  “Sinus Bradycardia. Heart rate is strictly less than 60 bpm.”
  “Rhythm is regular but significantly slow.”
  “Long P-P intervals.”
- Tachycardia
  “Sinus Tachycardia. Heart rate exceeds 100 bpm but the rhythm remains regular.”
  “Fast, steady beating with consistent but shortened P-P intervals.”
  “Distinct from chaotic rhythms.”

BioBERT generates a 768-dimensional [CLS] representation, which is subsequently projected into the shared 128-dimensional embedding space via a linear transformation:

(12) $$z_{s}=W_{\mathrm{proj}}h_{\mathrm{CLS}}+b_{\mathrm{proj}},\;W_{\mathrm{proj}}\in R^{128\times 768}\;$$

This embedding serves as the semantic anchor for rhythm-level supervision within the contrastive learning objective. The contrastive objective follows the InfoNCE formulation [20].

#### 2.9.4. InfoNCE Contrastive Loss

Wave–clinical alignment:

(13) $$L_{\mathrm{wave}↔\mathrm{clinical}}=-\frac{1}{N}\sum_{i=1}^{N}\log\left(\frac{\exp\left(\frac{\mathrm{sim}(z_{w}^{\left(i\right)},z_{c}^{\left(i\right)})}{\tau }\right)}{\sum_{j=1}^{N}\exp\left(\frac{\mathrm{sim}(z_{w}^{\left(i\right)},z_{c}^{\left(j\right)})}{\tau }\right)}\right)$$

Wave–state alignment:

(14) $$L_{\mathrm{wave}↔\mathrm{state}}=-\frac{1}{N}\sum_{i=1}^{N}\log\left(\frac{\exp\left(\frac{\mathrm{sim}(z_{w}^{\left(i\right)},z_{s}^{\left(i\right)})}{\tau }\right)}{\sum_{k=1}^{K}\exp\left(\frac{\mathrm{sim}(z_{w}^{\left(i\right)},z_{s}^{\left(k\right)})}{\tau }\right)}\right)$$

where

- sim(·,·) denotes cosine similarity.
- τ is the temperature parameter.
- N is the batch size.
- K is the number of rhythm classes.

Final contrastive loss:

(15) $$L_{{\mathrm{total}}_{2}}=\alpha \;L_{\mathrm{wave}↔\mathrm{clinical}}+\lambda \;L_{\mathrm{wave}↔\mathrm{state}}\;$$

This objective encourages waveforms to be close to both their corresponding clinical profiles and their rhythm-level semantic descriptions, resulting in a unified representation space that captures both patient-specific and physiological aspects of arrhythmia.

### 2.10. Multitask Model: U-Net with CLIP Encoder

**Figure 7:**
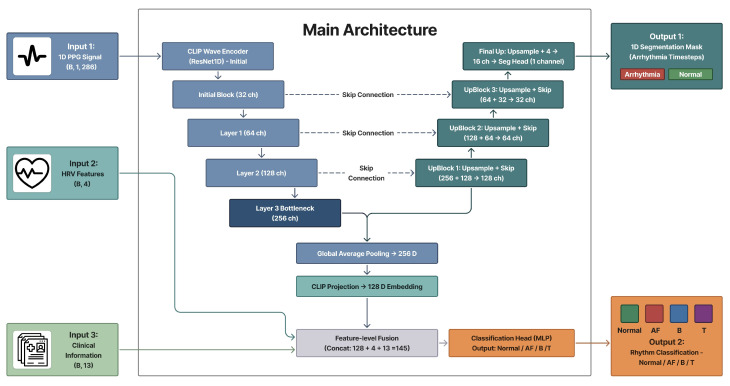
Proposed CLIP-initialized multi-task U-Net. The ResNet1D encoder pretrained with the CLIP-BioBERT framework acts as the U-Net encoder (blue blocks), and its bottleneck output is projected to a 128-dimensional CLIP embedding. HRV features and 13 structured clinical variables (green blocks) are concatenated with this embedding and passed to a classification head for four-class rhythm prediction, while the decoder path produces a 1D segmentation mask that localizes abnormal intervals.

The final proposed model reuses the pretrained CLIP waveform encoder as the encoder of a 1D U-Net (Figure 7). Encoder weights are initialized from CLIP pretraining; they are frozen for early stabilization and then fine-tuned following standard transfer learning practice [21].

#### 2.10.1. Encoder Initialization

The ResNet1D waveform encoder trained in the CLIP framework replaces the encoder blocks of the baseline U-Net. It consists of an initial convolutional stem followed by three residual blocks:

- An initial block with a 1×15 convolution (1 → 32 channels) and max pooling, reducing the temporal resolution from *L* to L/4.
- Layer 1: a residual block with 64 channels and stride 2 (output size L/8).
- Layer 2: a residual block with 128 channels and stride 2 (output size L/16).
- Layer 3: a residual block with 256 channels and stride 2 (output size L/32).

The feature maps at four scales {L/4,L/8,L/16,L/32} with channel dimensions {32,64,128,256} are exported as skip connections to the decoder, preserving multi-scale temporal information. The final bottleneck representation (256 channels) is passed through a linear projection layer to obtain the 128-dimensional CLIP embedding that was used during contrastive pretraining. During multitask training, these encoder and projection weights are initialized from the CLIP checkpoint; they are kept frozen for the first few epochs and then jointly fine-tuned with the rest of the network to stabilize optimization.

#### 2.10.2. Decoder and Segmentation Output

The decoder mirrors the encoder hierarchy using three upsampling blocks followed by a final refinement stage. Each upsampling block first increases the temporal resolution by a factor of two using linear interpolation, concatenates the corresponding encoder feature map along the channel dimension, and then applies a DoubleConv module (two successive Conv1D + GroupNorm + ReLU layers) to fuse information from encoder and decoder paths. Concretely, the channel dimensions evolve as corresponding to resolutions L/16, L/8, and L/4, respectively.

(16) 256+128→128,128+64→64,64+32→32

A final upsampling layer increases the resolution by an additional factor of four and a 1×1 convolution (32 → 16 channels) produces the last decoder feature map. The segmentation head then applies a 1×1 convolution to obtain a single-channel logit map $\hat{m}\in R^{B\times 1\times L}$, which is finally interpolated, if necessary, to exactly match the input length (286 samples in our experiments). Group Normalization is used throughout the decoder instead of Batch Normalization to ensure stable training under small effective batch sizes typical of high-resolution physiological waveforms.

#### 2.10.3. Classification Branch

For rhythm classification, global average pooling is applied to the bottleneck feature of the CLIP encoder to obtain a 256-dimensional waveform summary vector, which is then projected to the 128-dimensional CLIP embedding $z_{w}$ via the pretrained projection layer. This embedding is concatenated with two additional modalities:

- The HRV feature vector (4 dimensions).
- The 13-dimensional vector of normalized clinical variables.

The resulting 145-dimensional multimodal feature is passed through a multilayer perceptron consisting of two hidden layers (145 → 128 → 64) with Layer Normalization, ReLU activations, and dropout regularization, followed by a final linear layer that outputs logits for the four rhythm classes (Normal, AF, B, and T). Thus, the classification head jointly exploits CLIP-initialized waveform features, beat-to-beat variability, and structured clinical context.

#### 2.10.4. Multitask Loss

The network is trained in a multitask manner with coupled classification and segmentation objectives. Let $\hat{y}\in R^{B\times 4}$ denote the rhythm logits and let $\hat{m}\in R^{B\times 1\times L}$ denote the segmentation logits. For rhythm classification, we employ a focal loss $L_{\mathrm{focal}}$ with class-dependent weights to mitigate label imbalance and emphasize hard examples. For segmentation, we use a combination of sigmoid cross-entropy and Dice loss:

(17) $$L_{\mathrm{seg}}=\frac{1}{2}\;L_{\mathrm{BCE}}\left(\hat{m},m\right)+\frac{1}{2}\;L_{\mathrm{Dice}}\left(\hat{m},m\right)\;$$

where *m* is the ground-truth abnormal interval mask. The overall training objective is

(18) $$L_{\mathrm{total}}=\alpha \;L_{\mathrm{focal}}+\beta \;L_{\mathrm{seg}}\;$$

with α=β=1 in all experiments, reflecting equal importance assigned to accurate rhythm classification and temporal localization of arrhythmias.

### 2.11. LLM-Based Explainability via Retrieval-Augmented Clinical Reasoning

Deep learning models for physiological signal analysis often provide high accuracy but lack interpretability, limiting trust and real-world adoption in clinical settings. To address this gap, we integrated a Retrieval-Augmented Generation (RAG) [12] pipeline using a large language model (LLM) to generate clinically grounded, guideline-supported explanations for each prediction. The LLM module operates on multimodal inputs—PPG-derived waveform images, clinical metadata, similar-patient embeddings, and model outputs—and produces structured diagnostic narratives resembling human clinical reasoning.

#### 2.11.1. LLM Model and Inference Mode

We integrated a large language model (LLM) as a post-hoc explainability module to generate concise, guideline-grounded narrative reports from multimodal evidence. In this work, the LLM is used only for report generation and interpretation and does not participate in training the arrhythmia detection model. We used a Gemini family multimodal model (Flash version) through an API interface [22].

This selection was motivated by (i) its native multimodal capability for waveform-visual evidence, (ii) low-latency responses suitable for interactive analysis, and (iii) sufficient context length for retrieval-augmented prompting.

The LLM was used in a zero-shot manner; no fine-tuning or additional training was performed on our dataset. This design choice was made because (i) the arrhythmia cohort size is limited, making LLM fine-tuning prone to overfitting and unverifiable memorization, and (ii) our primary objective is to produce transparent, reproducible explanations strictly grounded on retrieved evidence rather than learned free-form generation.

The LLM was not trained, fine-tuned, or adapted on VitalDB or any patient-specific data in this study. All outputs are generated from provided evidence (model predictions, retrieved references, and waveform visualization) under a constrained prompt template.

#### 2.11.2. Evidence Inputs to the LLM

The LLM receives four categories of evidence:

1. Model outputs: Predicted rhythm class probabilities, confidence scores, and the 1D segmentation mask that localizes abnormal intervals.
2. Waveform visual evidence: A rendered plot of the PPG segment with segmentation overlay (mask-highlighted abnormal region), provided as an image input to the multimodal LLM.
3. Patient context: Structured clinical variables and extracted HRV features (Heart Rate, SDNN, RMSSD, and pNN50).
4. Retrieved references (RAG): (a) Guideline excerpts and (b) similar-patient summaries retrieved from our embedding index (Section 2.11.3).

#### 2.11.3. Retrieval Mechanism (RAG)

To reduce hallucination and ensure guideline consistency, we adopt Retrieval-Augmented Generation (RAG). Given a query case, we compute an embedding from structured clinical variables using the same clinical encoder employed in contrastive pretraining. We then retrieve top-*k* nearest neighbors from the training database using cosine similarity:

(19) $$\mathrm{sim}\left(z_{q},z_{i}\right)=\frac{z_{q}\cdot z_{i}}{∥z_{q}∥∥z_{i}∥}$$

Retrieved cases provide empirical context (e.g., label distribution among neighbors) that is injected into the prompt as structured evidence.

#### 2.11.4. Prompt Template and Decoding Parameters

We used a structured prompt template to constrain generation and improve reproducibility. The prompt explicitly includes: (i) predicted class and confidence, (ii) HRV summary, (iii) abnormal interval duration and localization, (iv) retrieved similar-case statistics, and (v) guideline excerpts. The LLM is instructed to (a) avoid adding information not present in the evidence, (b) cite only the provided guideline statements, and (c) output a short structured report with fixed headings.

Decoding was configured to prioritize consistency over creativity (low temperature). In all experiments, we used deterministic or low-stochasticity settings (e.g., temperature ≤0.3) and bounded maximum output length to prevent overly verbose responses.

#### 2.11.5. Reliability and Safety Guardrails

Because LLM outputs may contain hallucinated or overconfident statements [23], we implemented guardrails:

- Evidence injection: the model is restricted to retrieved guideline excerpts and case summaries, without access to open-ended external web knowledge.
- Structured output constraint: outputs are limited to predefined sections (Diagnosis, Evidence, Interpretation, and Recommendations).
- Consistency check: a secondary verification prompt is optionally applied to detect contradictions between the output and the provided evidence.

Importantly, the generated report is intended as an assistive explanation rather than a clinical decision.

### 2.12. Streamlit Web Implementation

To demonstrate the clinical feasibility of the proposed framework, we developed a web-based diagnostic support platform. The system, implemented using Python (v3.10) and Streamlit (v1.32.0), functions as a comprehensive end-to-end solution by integrating user data management, real-time waveform analysis, and LLM-driven reporting.

#### 2.12.1. System Architecture

The application is structured into three primary modular components to ensure scalability and maintainability:

- Frontend and Orchestration: This module manages the user interface (UI), handles user inputs, and orchestrates overall data flow. It coordinates the interaction between deep learning models and databases, ensuring seamless state management across user sessions.
- Inference Engine: This backend module hosts the pre-trained CLIP-initialized multi-task U-Net. It is responsible for loading model weights, processing input tensors, and generating classification probabilities and segmentation masks.
- LLM Utilities: This module manages the Retrieval-Augmented Generation (RAG) pipeline. It handles the encoding of clinical queries, retrieval of similar patient cases from the vector index, and the construction of prompts for the Gemini API to generate diagnostic reports.

#### 2.12.2. User Interface and Workflow

**Figure 8:**
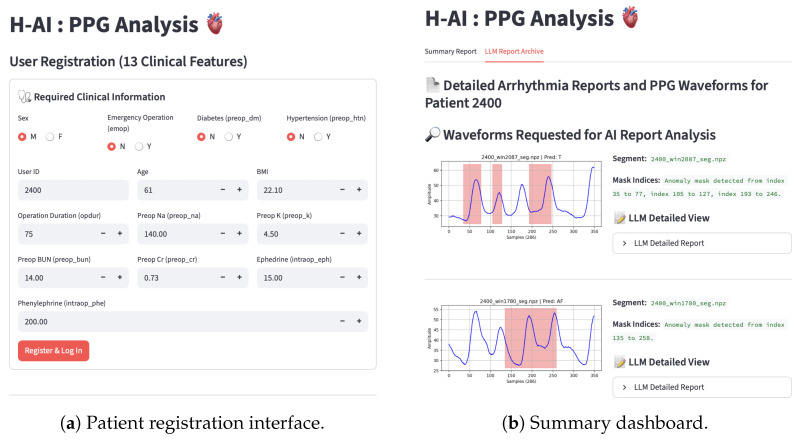
User interface for patient data management and aggregate analysis. (**a**) The registration form for inputting 13 clinical variables (e.g., demographics, electrolytes), which are essential for the clinical encoder. (**b**) The arrhythmia summary dashboard providing the distribution of predicted rhythms and retrieving relevant medical guidelines via the RAG framework.

- Patient Registration and Authentication: When accessing the system, users are required to complete a registration process by providing a unique Patient ID and 13 clinical variables (e.g., age, sex, BMI, preoperative electrolyte levels, and intraoperative vasopressor administration) (Figure 8a). These variables are indispensable for normalizing the input to the clinical encoder and to construct a physiologically meaningful patient profile within the RAG-based retrieval framework.
- Data Ingestion and Preprocessing: Users upload PPG signal files directly through the interface. The app.py module automatically preprocesses these raw signals—resampling, normalizing, and formatting them into tensors compatible with the inference engine.
- Real-Time Inference and Visualization: The system processes the uploaded segments sequentially. The results are visualized in an interactive dashboard that categorizes segments by rhythm type (Normal, AF, Bradycardia, Tachycardia) using distinct tabs (Figure 8b). For each segment identified as arrhythmic, the system displays the waveform with a semi-transparent overlay indicating the predicted abnormal interval (segmentation mask) and the model’s confidence score.

#### 2.12.3. On-Demand LLM Reporting Pipeline

**Figure 9:**
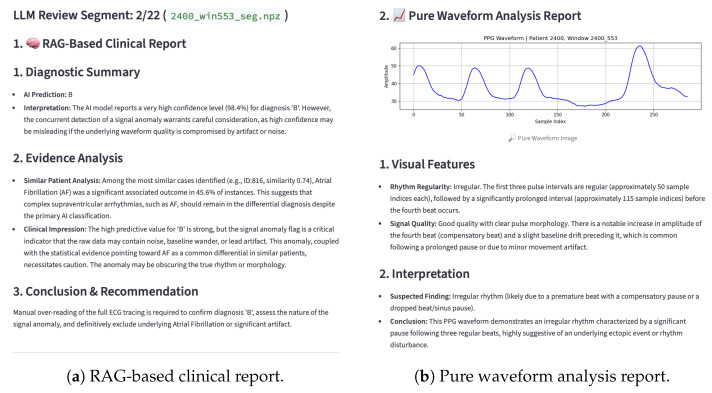
The on-demand “Deep Diagnosis” reporting interface. (**a**) RAG-based clinical report: the system retrieves similar patient cases from the vector database to provide statistical evidence (e.g., “AF was a significant outcome in 45.6% of similar instances”) and clinical impressions. (**b**) Pure waveform analysis: the vision-capable LLM analyzes the morphological features of the signal image, identifying rhythm irregularities and signal quality issues (e.g., baseline drift, compensatory pauses) to support the final diagnosis.

To optimize computational resources and API costs, the “Deep Diagnosis” feature is implemented as an on-demand service rather than an automatic process for every segment. When a clinician selects a specific abnormal segment for deeper analysis, the system triggers a two-stage reporting process (Figure 9):

- RAG-Based Clinical Report: The system retrieves similar patient cases based on the clinical embeddings and generates a report comparing the current patient’s profile with historical aggregate data.
- Pure Waveform Analysis Report: The system passes the waveform image to the vision-capable LLM to analyze morphological features (e.g., rhythm regularity, signal quality).

#### 2.12.4. Database and Optimization

All processed data—including raw waveforms, predicted masks, and generated LLM reports—are stored in an SQLite database. This transformation from static file loading to a dynamic database-driven architecture enables retrospective auditing, allowing clinicians to review a “Report Archive” for longitudinal patient monitoring.

## 3. Results

This section presents quantitative and qualitative evaluation of the proposed framework, including arrhythmia classification, segmentation performance, training dynamics, the effect of CLIP-BioBERT pretraining, and examples of LLM-generated clinical reports. All evaluations were conducted on the test set containing five patients with mixed normal and abnormal segments (386 samples).

### 3.1. Evaluation Metrics

For classification, we report the following:

- F1-score (per class);
- Accuracy;
- Precision;
- Recall;
- Confusion matrix.

For segmentation, we compute the following:

- Pixel accuracy;
- Dice similarity coefficient (DSC);
- Intersection over Union (IoU) [18].

### 3.2. Overall Classification Performance

The classification performance of the proposed model and the U-Net baseline on the test set is summarized in Table 2. Overall, the proposed model demonstrated superior performance in terms of accuracy and most class-specific F1-scores compared to the baseline.

The proposed model achieved an overall accuracy of 0.9119, which is an absolute improvement of 4.92 percentage points over the U-Net baseline (0.8627). In terms of class-specific F1-scores, the proposed model consistently outperformed the baseline for the majority of arrhythmia types:

- Normal: The F1-score increased from 0.9452 to 0.9775.
- Atrial Fibrillation (AF): A notable improvement was observed, with the F1-score rising from 0.7940 to 0.8352.
- Bradycardia (Brady): The most significant gain was achieved in this category, with the F1-score improving by 11.6% (from 0.7778 to 0.8681).

However, a significant performance degradation was observed in the Tachycardia (Tachy) category. While the U-Net baseline recorded an F1-score of 0.6000, the proposed model’s performance dropped sharply to 0.2857. The apparent performance degradation in the Tachycardia class should be interpreted with caution, as only a few samples were available for this category. Therefore, the observed decrease is more likely due to extreme class scarcity rather than a systematic limitation of the proposed architecture.

### 3.3. Detailed Class Metrics

To gain a more granular understanding of the proposed model’s performance, detailed per-class metrics, including precision and recall, are presented in Table 3. The analysis reveals distinct performance characteristics for each arrhythmia type.

The model demonstrated exceptionally high and balanced performance for the Normal class, achieving both precision and recall above 0.97. For Atrial Fibrillation (AF), the model exhibited a high recall of 0.9500, indicating its effectiveness in capturing the majority of AF cases, which is critical for clinical screening. However, the relatively lower precision of 0.7451 suggests a tendency towards false positive detections in AF identification. In the case of Bradycardia, the model maintained a high precision of 0.9405 with a reasonable recall of 0.8061. The most notable observation is found in the Tachycardia class. While the model achieved a perfect precision of 1.0000—meaning every instance it labeled as Tachycardia was correct—the recall was significantly low at 0.1667. This imbalance suggests that the model is extremely conservative in detecting Tachycardia, potentially due to a severe class imbalance in the training data or highly specific feature requirements for this category. These findings underscore the need for targeted data augmentation or loss-weighting strategies to improve the sensitivity of Tachycardia detection in future work.

### 3.4. Confusion Matrix Analysis

**Figure 10:**
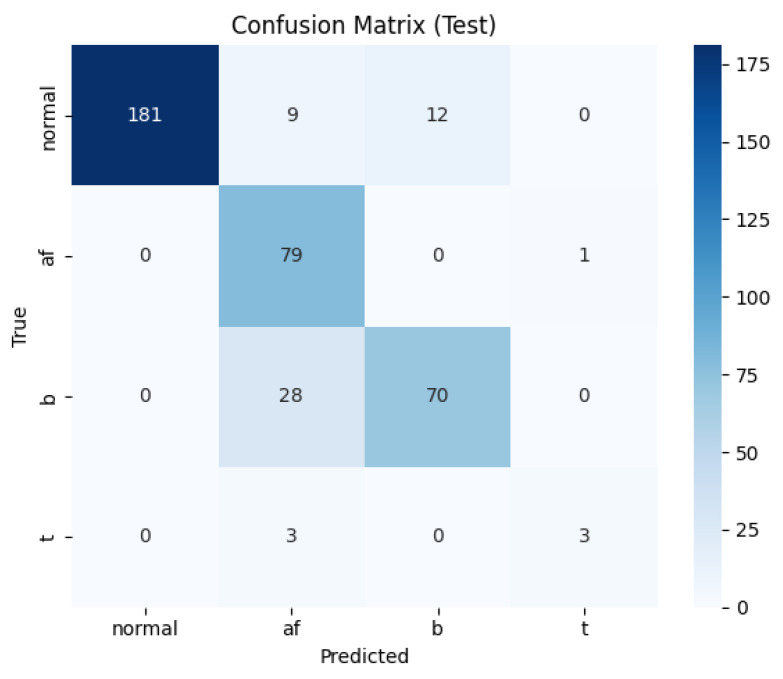
Confusion matrix of the baseline multi-task U-Net on the test set. Axes: true label (y) vs. predicted label (x).

**Figure 11:**
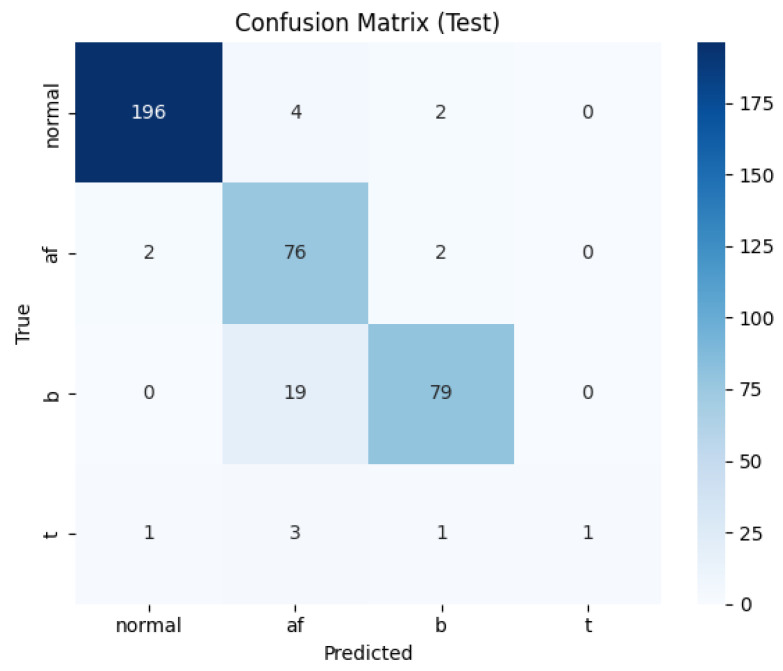
Confusion matrix of the proposed U-Net + CLIP model on the test set. Axes: true label (y) vs. predicted label (x).

To further evaluate classification reliability, the confusion matrices for the baseline U-Net and the proposed U-Net + CLIP model were compared (Figure 10 and Figure 11). The proposed model demonstrated a significant reduction in inter-class confusion, particularly between Normal and arrhythmic conditions.

In the Normal class, the proposed model correctly identified 196 cases, an improvement from 181 in the baseline. Notably, misclassifications of Normal signals as Bradycardia were reduced from 12 to two, and as AF from nine to four. This indicates that the integration of CLIP features enhances the model’s ability to distinguish subtle morphological variations in heart rate signals.

For Bradycardia, the baseline model frequently confused it with AF (28 cases), whereas the proposed model reduced this error to 19 cases, leading to an increased number of true positives. Regarding Atrial Fibrillation (AF), the model maintained a high sensitivity (Recall: 0.9500), which is crucial for clinical diagnosis, although 19 cases of Bradycardia were still misclassified as AF.

The Tachycardia class remains a challenge. The confusion matrix (Figure 11) reveals that only six Tachycardia samples were present in the test set, with the model correctly identifying only one. The majority of Tachycardia cases were misclassified as AF (three cases). This suggests that the severe class imbalance in the dataset, combined with the morphological similarities between certain Tachycardia and AF segments, limits the model’s sensitivity for this specific category.

### 3.5. Segmentation Performance

The segmentation performance was evaluated using Pixel Accuracy, Dice Score, and Intersection over Union (IoU) to determine the model’s ability to precisely localize arrhythmia segments within the signals. As summarized in Table 4, the proposed model significantly outperformed the U-Net baseline across all evaluation metrics.

The proposed model achieved a Pixel Accuracy of 0.9344, representing a 4.0% improvement over the baseline’s 0.8983. More substantial gains were observed in the overlap-based metrics:

- Dice Score: The proposed model reached 0.7167, a relative improvement of 23.2% compared to the baseline (0.5815).
- Intersection over Union (IoU): The IoU score increased from 0.4213 to 0.5902, marking a 40.1% relative improvement.

These results indicate that while the baseline U-Net can identify general regions of interest, the integration of the proposed architecture—likely benefiting from global feature alignment—allows for much sharper and more accurate boundary detection of arrhythmic episodes. The marked increase in IoU, in particular, suggests that the proposed model is more robust against false positive segmentation, which is a common challenge in processing complex physiological waveforms like PPG.

### 3.6. Example Results

**Figure 12:**
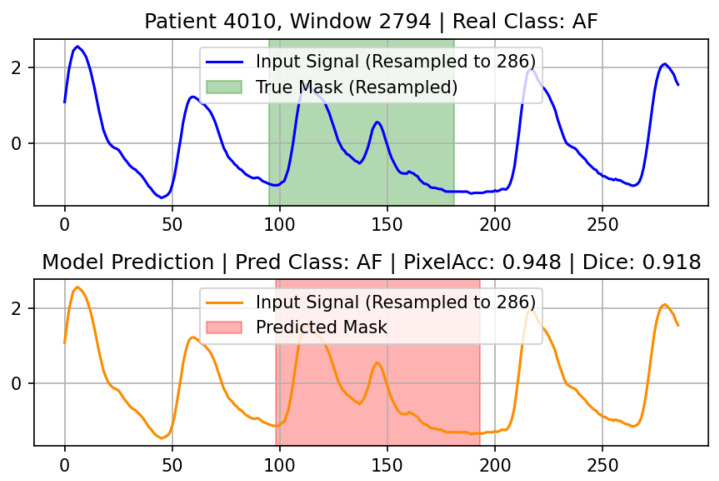
Example AF case correctly classified by the proposed model. x-axis: sample index (0–286) corresponding to resampled time; y-axis: normalized amplitude.

**Figure 13:**
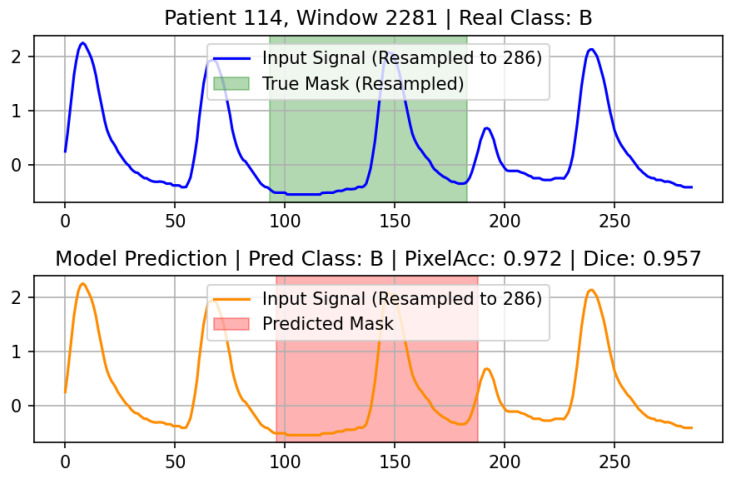
Example Bradycardia case correctly classified and segmented. x-axis: sample index (0–286) corresponding to resampled time; y-axis: normalized amplitude.

To qualitatively evaluate the model’s segmentation and classification capabilities, we visualized specific prediction instances from the test set. Figure 12 illustrates a representative example of an Atrial Fibrillation (AF) case that was correctly classified and precisely segmented by the proposed model. Similarly, Figure 13 demonstrates an accurately localized and classified Bradycardia segment. These visualizations confirm that the multi-task architecture effectively captures the distinct temporal boundaries and morphological irregularities associated with different arrhythmic events.

### 3.7. Ablation Study

The ablation study results in Table 5 confirm the contribution of each module. Starting from a baseline U-Net accuracy of 0.8254, adding clinical data improved accuracy to 0.8541 (+2.87%). The addition of HRV features alone (U-Net + HRV) yielded an even higher accuracy of 0.8627, suggesting that signal-derived variability features provide more discriminative information than basic clinical data in this task.

However, the most substantial leap in performance occurred with the full proposed model, which incorporates CLIP-based multimodal fusion. This configuration achieved an accuracy of 0.9119 and a Dice score of 0.7167. The significant jump in Dice score (+13.52% over HRV-only) highlights the efficacy of CLIP in aligning semantic clinical descriptors with physiological signals, thereby enabling the model to learn more robust features for precise arrhythmia segmentation.

## 4. Discussion

### 4.1. Clinical Significance of PPG-Based Multitask Modeling

Our results show that a PPG-based system can provide clinically relevant multi-class arrhythmia detection with temporal localization and interpretability, extending prior AF-focused wearable studies [4]. Improvements in Table 2 and Table 4 indicate that multimodal pretraining and fusion benefit challenging classes, particularly Atrial Fibrillation (AF) and Bradycardia.

### 4.2. Impact of CLIP-BioBERT Pretraining

The CLIP-BioBERT module improves both classification and segmentation by aligning waveform morphology with clinical context and semantic rhythm descriptions [9]. This alignment yields a more structured representation space that stabilizes downstream training.

### 4.3. Interpretability Through RAG–LLM

The RAG–LLM module generates guideline-grounded narratives by combining model outputs, retrieved similar cases, and explicit guideline snippets, while using a safety layer (error-check prompt) to reduce contradictions. Because large language models may produce hallucinated or overconfident statements [23], the generated content is intended for decision support and requires expert review.

### 4.4. Limitations

This study has several limitations. First, our experiments were conducted using a publicly available perioperative dataset [14], and no additional prospective or multi-center clinical data were collected in this work. Therefore, external validity across institutions, devices, and real-world ambulatory settings (e.g., wrist-worn reflectance PPG) remains to be established.

Second, although the proposed framework provides LLM-generated narrative reports grounded in retrieved evidence, the clinical validity of these explanations was not formally evaluated by board-certified cardiologists in this study. Accordingly, the generated reports should be interpreted as assistive summaries rather than definitive clinical conclusions. Future work will include expert review protocols and clinical user studies to assess consistency with cardiological practice and to quantify trustworthiness and utility in workflows.

Third, a major limitation lies in the inherent class imbalance in the dataset, particularly the extreme scarcity of Tachycardia (T) samples. While the CLIP-U-Net model demonstrated high performance for prevalent categories such as ‘Normal’ and ‘AF,’ the limited number of Tachycardia cases in the test set may constrain the statistical generalizability of the performance metrics for this specific class. Consequently, the current results for infrequent arrhythmia types should be regarded as preliminary. Future research should prioritize the integration of more balanced datasets or the application of advanced data augmentation and generative techniques to ensure robust and reliable detection across all arrhythmia subtypes.

### 4.5. Future Work

Several important research directions remain to further strengthen the clinical validity, generalizability, and practical deployment of the proposed framework.

- Multi-Center and Device-Level Validation: The present study relies exclusively on perioperative data from the VitalDB database. Although this dataset provides high-quality intraoperative signals, waveform characteristics may vary across institutions, monitoring devices, and acquisition protocols. Future work will involve external validation using multi-center datasets and heterogeneous monitoring systems, including different bedside monitors and wearable-grade sensors. Cross-device evaluation will be essential to quantify domain shift and assess the robustness of the learned multimodal representations.
- Wearable-Domain Adaptation: The current framework was developed using transmission-mode intraoperative PPG signals obtained under general anesthesia. In contrast, ambulatory and consumer-grade wearable devices typically use reflectance-mode PPG under motion-rich environments. Future studies will investigate domain adaptation techniques, including adversarial feature alignment, self-supervised pretraining on large-scale wearable datasets, and fine-tuning strategies tailored for motion-corrupted signals. Such adaptation is critical for extending the framework to remote and home-based monitoring applications.
- Prospective Clinical Evaluation and Cardiologist Review: Although the LLM-based reports are guideline-grounded and retrieval-constrained, their clinical usefulness has not yet been systematically evaluated. Future work will include blinded evaluation by board-certified cardiologists to assess diagnostic consistency, interpretability, and workflow integration. Quantitative measures such as agreement rate, clinical usefulness scoring, and error categorization will be incorporated to rigorously evaluate the reliability of AI-generated explanations.
- Expansion of Arrhythmia Taxonomy: This study focuses on four rhythm classes (Normal, AF, Bradycardia, and Tachycardia). However, clinically significant arrhythmias such as atrial flutter, premature atrial contractions (PACs), premature ventricular contractions (PVCs), and supraventricular tachycardia (SVT) are not included. Future extensions will expand the classification taxonomy and explore hierarchical classification strategies to differentiate subtle rhythm subclasses.
- Improved Handling of Class Imbalance: The scarcity of Tachycardia samples highlights the need for more balanced datasets. Future work will explore synthetic data generation using generative adversarial networks (GANs), diffusion-based signal synthesis, and class-aware curriculum learning strategies to improve sensitivity for rare arrhythmia types.
- Lightweight and Edge Deployment: For real-world clinical or wearable integration, computational efficiency is essential. Future studies will investigate model compression techniques such as knowledge distillation, structured pruning, low-rank approximation, and quantization-aware training. These methods will enable deployment on edge devices or embedded systems while maintaining acceptable diagnostic accuracy.
- Beyond Post-Hoc Explainability: Currently, explainability is provided through a post-hoc RAG–LLM pipeline. Future work will explore intrinsically interpretable architectures, attention-based attribution mechanisms, and physiological feature disentanglement to bridge the gap between learned representations and clinically recognizable waveform characteristics.

Together, these directions aim to transform the proposed framework from a proof-of-concept perioperative study into a scalable, clinically validated, and deployable multimodal arrhythmia monitoring system.

## 5. Conclusions

We presented an integrated multimodal and explainable PPG arrhythmia detection framework leveraging CLIP-style contrastive learning, multitask U-Net segmentation/classification, and Retrieval-Augmented LLM reporting. The proposed system improves both classification accuracy and segmentation Dice on the VitalDB dataset and demonstrates the feasibility of combining waveform modeling with clinical context and guideline-grounded narrative generation. While further multi-center validation and expert clinical evaluation are required, the framework illustrates a scalable direction for interpretable PPG-based arrhythmia analysis in perioperative and remote monitoring settings.

## Figures and Tables

**Table 1:** PPG acquisition characteristics used in this study (VitalDB). When a parameter is not explicitly specified in VitalDB metadata, we report standard intraoperative pulse oximetry characteristics based on established clinical practice.

Parameter	Value/Description
Measurement site	Finger probe (typical intraoperative pulse oximetry) [3]
Data source	VitalDB perioperative waveform database [14]
Monitoring system	Bedside monitor (Philips IntelliVue series)
Recording environment	Intraoperative monitoring under general anesthesia
Working mode	Transmission-mode pulse oximetry (typical clinical monitoring)
Original record length	Approximately 9–10 min per patient record (variable by case)
Sampling frequency (this study)	100 Hz (downsampled for model input)
Signal amplitude unit	Device-dependent arbitrary units (a.u.)
Primary windowing (this study)	60 s windows (6000 samples at 100 Hz)

**Table 2:** Classification performance comparison on the test set.

Model	Accuracy	Normal F1	AF F1	Bradycardia F1	Tachycardia F1
U-Net baseline	0.8627	0.9452	0.7940	0.7778	0.6000
Proposed model	**0.9119**	**0.9775**	**0.8352**	**0.8681**	0.2857

**Table 3:** Detailed per-class metrics on the test set (proposed model).

Class	Precision	Recall	F1
Normal	0.9849	0.9703	0.9775
Atrial Fibrillation (AF)	0.7451	0.9500	0.8352
Bradycardia	0.9405	0.8061	0.8681
Tachycardia	1.0000	0.1667	0.2857

**Table 4:** Segmentation results for baseline and proposed models.

Model	Pixel Accuracy	Dice	IoU
U-Net baseline	0.8983	0.5815	0.4213
Proposed model	**0.9344**	**0.7167**	**0.5902**

**Table 5:** Ablation results on classification accuracy and Dice score.

Configuration	Accuracy	Dice
U-Net (no HRV, no clinical)	0.8254	0.5532
U-Net + clinical only	0.8541	0.5795
U-Net + HRV only	0.8627	0.5815
U-Net + CLIP + HRV + clinical (Proposed Model)	**0.9119**	**0.7167**

## Data Availability

The dataset analyzed in this study is publicly available at the VitalDB repository (https://vitaldb.net, accessed on 8 December 2025). Additional code and resources used in model implementation are available from the corresponding author upon reasonable request.

## Abbreviations

PPG	Photoplethysmography
ECG	Electrocardiography
AF	Atrial Fibrillation
B	Bradycardia
T	Tachycardia
HRV	Heart Rate Variability
HR	Heart Rate
SDNN	Standard Deviation of NN intervals
RMSSD	Root Mean Square of Successive Differences
U-Net	U-shaped Convolutional Neural Network
CLIP	Contrastive Language–Image Pretraining

BioBERT	Biomedical Bidirectional Encoder Representations from Transformers
LLM	Large Language Model
RAG	Retrieval-Augmented Generation
SE	Squeeze-and-Excitation
IoU	Intersection over Union
DSC	Dice Similarity Coefficient
MLP	Multilayer Perceptron
